# Anxiety and depression among Chinese adolescents during the COVID-19: an overestimation of the problem

**DOI:** 10.1038/s41398-021-01748-2

**Published:** 2021-12-09

**Authors:** Lara Kojok, Katie Bodenstein, Marjolaine Rivest-Beauregard, Quinta Seon, Ram P. Sapkota, Alain Brunet

**Affiliations:** 1 Department of Psychiatry, McGill University, Montréal (Québec), Montréal, QC Canada; 2 Douglas Mental Health University Institute, Montréal (Québec), Verdun, QC Canada; 3 Department of Psychology, University of Regina, Saskatchewan, Regina, SK Canada

**Keywords:** Depression, Human behaviour

**DEAR EDITOR**,

We read with interest the article by Chen and colleagues [1] which aimed to examine changes in depression and anxiety among Chinese adolescents during and after the initial COVID-19 outbreak. These authors found that the prevalence of anxiety and depression significantly increased in Chinese adolescents after the initial outbreak. We commend the authors for addressing a pressing topic. However, we would call into question the validity of the results, and, further, the integral purpose of the article.

First, we believe the study design is not appropriate and poses several problems. This study involved the completion of two surveys: an initial survey completed by a sample of 9554 people and a second survey by 10,605 individuals. However, participants who completed both surveys (*n* = 6719) were removed. It is unclear why the authors decided to exclude such a large number of participants. Indeed, the rationale was not provided as to why they omitted the opportunity to conduct a longitudinal study using the two time points of collected data, rather than conducting two cross-sectional studies, which they opted for in this paper. Doing so would have allowed for more robust conclusions, as each participant would serve as their own control. In addition, within-subject designs are considered superior as they have greater statistical power [2].

In addition, excluding participants who completed both surveys also raises the question of statistical difference between the initial samples and the samples used in the analyses, after the exclusion of duplicate participants. Were these compared for the statistical difference? If so, why weren’t the results reported?

Furthermore, Chen and colleagues [1] conclude that compared to the initial outbreak, “the prevalence of depression and anxiety significantly increased after the initial COVID-19 outbreak had remitted.” However, the authors are comparing two different groups of people. Thus, stating that rates “increased” is problematic: rates may be higher, but the extrapolation of increased rates when examining two different sets of people is unwarranted. The authors treat the results as if they were a longitudinal study with temporal continuity. Besides, snowball sampling may be appropriate to examine relationships, but what about ‘rates’? This is inappropriate.

Moreover, the authors present “prevalence” and “rates” of disorders (i.e., depression and anxiety), but this study exclusively used self-report measures as opposed to diagnostic interviews. The authors fail to report that self-report measures may not be used to diagnose disorders; further, they may substantially overestimate prevalence [3]. As such, caution should be employed when utilizing such terms as they would mislead readers. In addition, the cut-off scores the authors used for their measures (i.e., the Center for Epidemiologic Studies Depression Scale; CES-D, and the General Anxiety Disorder-7 scale; GAD-7) are extremely lenient and may result in inflation of the reported “rates” of anxiety and depression. For instance, the cut point of 4 on the GAD-7 was used in this study to identify adolescents with anxiety, whereas the original paper stated that a score of 10 or higher represents an adequate cut point for the identification of GAD-7 cases [4]. Other papers validating the GAD-7 among Chinese people with epilepsy and Chinese pregnant women utilized cut-off scores of 6 and 7 respectively [5, 6]. Similarly, a cut-score of 15 on the CES-D was utilized to identify adolescents with depression in this study, whereas a systematic review of 28 studies proposed an optimal cut point of 20 [7]. Therefore, cut-off scores in this study appear to be too low, on top of self-report measures already overestimating the prevalence, leading to a ringing of false alarms.

Further, we cannot help but notice that several elements are missing from the methods section. Indeed, there was no mention of how anything other than anxiety and depression was measured (i.e., the correlates of anxiety and depression that they explored). Measures of sleep duration, study duration, exercise duration, study efficiency, being concerned about entering a higher grade, are all absent.

All the points mentioned above bring into question the validity of their findings that appear quite an alarmist. Moreover, numerous other questions come up as we go through the paper. Importantly, were the assumptions for regression analyses tested? If so, why weren’t they reported? Further, how did they manage data from participants who did not complete the totality of the survey (i.e., was the data discarded or corrected for)? What was the minimum completion rate accepted for participants to be included in the analyses? Additionally, it is stated in the paper that 46% of the initial sample were males, but what was the percentage of males in the final sample that was used in the analyses (i.e., after excluding participants who completed both surveys)? These questions stack up with the more fundamental problems in this paper.

Finally, we were wondering what the findings would have been, had different cut-off scores been used, and had a longitudinal design been used. We highly encourage the authors to re-run their analyses with these considerations in mind, or to make their data accessible for others to do so.

In sum, this paper tackled an important topic and a population that was heavily affected by the pandemic and whose mental health warrants investigation. However, considering the limitations, the results may be a misrepresentation of reality and an inflation of the gravity of the problem suggested.
